# Quantitative grip force assessment of muscular weakness in chronic inflammatory demyelinating polyneuropathy

**DOI:** 10.1186/s12883-019-1339-x

**Published:** 2019-06-08

**Authors:** Juliane Klehmet, Svenja Beutner, Sarah Hoffmann, Matthias Dornauer, Friedemann Paul, Ralf Reilmann, Alexander U. Brandt, Andreas Meisel

**Affiliations:** 1 NeuroCure Clinical Research Center, Charité - University Medicine Berlin, Charitéplatz 1, 10117 Berlin, Germany; 2 Department of Neurology, Jüdisches Krankenhaus Berlin, Heinz-Galinski-Strasse 1, 13347 Berlin, Germany; 3 George-Huntington-Institute, Technology-Park, Johann-Krane Weg 27, 48149 Muenster, Germany; 4 Department of Radiology, University Medicine of Muenster, Albert-Schweitzer Campus 1, 48149 Muenster, Germany; 5 Department of Neurodegenerative Diseases and Hertie-Institute for Clinical Brain Research, University of Tuebingen, Hoppe-Seyler Str. 3, 72076 Tuebingen, Germany

**Keywords:** CIDP, Clinical outcome parameter, CIDP subgroups, Case control studies, Diagnostic test assessment

## Abstract

**Background:**

In patients suffering from Chronic Inflammatory Demyelinating Polyneuropathy (CIDP) disease severity is assessed by Medical Research Counsil (MRC) Scale or Inflammatory Neuropathy Cause and Treatment (INCAT) disability score. However, none of these methods is appropriate to objectively assess muscle weakness or to detect very small subclinical changes. More objective and quantitative measures are needed in order to evaluate treatment efficiency or to detect subclinical affection of upper limps for early diagnosis. The goal of our study was to objectively quantify muscular weakness in CIDP patients with the non-invasive Quantitative Motor (Q-Motor) test of Grip Force Assessment (QGFA) as well as the Involuntary Movement Assessment (QIMA) and to search for differences between typical and atypical CIDP variants. In addition, we hypothesized that Q-Motor findings correlate with disease severity scales such as MRC or INCAT score.

**Methods:**

In this cross-sectional exploratory proof-of-concept study subjects with confirmed diagnosis of typical or atypical CIDP were examined and compared to healthy controls (HC). For Q-Motor tests all subjects had to lift a device (250 g and 500 g) equipped with an electromagnetic sensor that measured grip force (GF) and three-dimensional changes in position and orientation. The measures “grip force variability” (GFV), “position index” (PI) and “orientation index” (OI) were provided to assess involuntary movements due to muscular weakness.

**Results:**

33 patients with CIDP and 28 HC were included. All measures were significantly elevated in CIDP patients for both devices in the right and left hand compared to healthy controls. Subgroup analysis revealed no differences between typical and atypical CIDP variants. INCAT score only weakly correlated with OI and PI. However, there was a stronger correlation between MRC and QIMA parameters in both hands.

**Conclusion:**

Q-Motor assessments were capable to objectively assess muscular weakness in CIDP. In particular, QIMA measures detected subclinical generalized muscle weakness even in patients with milder disability. Sensitivity and rater-independence of Q-Motor assessments support a further exploration of QIMA measures as potential endpoints for future clinical trials in CIDP.

## Background

Chronic inflammatory demyelinating polyneuropathy (CIDP) is a rare autoimmune disorder of the peripheral nervous system leading to both proximal and distal weakness of all extremities. Beside this typical manifestation, there is a proportion of patients suffering from atypical variants such as pure sensory (sensory CIDP), distally acquired demyelinating polyneuropathy (DADS) or asymmetric multifocal acquired demyelinating sensory and motor polyneuropathy (MADSAM) neuropathies [1, 2]. CIDP is a demyelinating disease leading to typical demyelinating changes, which can be detected by nerve conduction studies according to different established demyelinating criteria (European Federation of Neurological Societies, EFNS; American Academy of Neurology, AAN) and by MR-neurography or ultrasound. However, diagnosis may still be challenging due to the great fraction of atypical variants for which no clear diagnostic criteria exist [3]. Moreover, patients may have suffered from the disease for long so that secondary axonal degeneration occurred. In these cases, established paraclinical criteria may not be sufficient alone. Accordingly, there is a remarkable rate of misdiagnosed CIDP, mainly atypical CIDP [4]. Recent studies revealed the presence of autoantibodies to paranodal junction proteins, such as neurofascin 155 and contactin 1 in a subpopulation of CIDP patients [5]. There is evidence that the underlying mechanism is distinct from those with classical macrophage-induced demyelination [6].

Besides diagnostic challenges, the assessment of muscular weakness is the most important treatment outcome parameter. Based on recent studies there is an increasing need to objectively assess the treatment efficacy in order to avoid overtreatment and reduce high treatment costs such as by intravenous immunoglobulins (IVIG). The *Medical Research Council Sum Score* is often applied in order to measure the muscle strength on the level of *impairment* [7], whereas the INCAT overall disability scale measures the disability resulting from muscular weakness [8]. However, none of these methods is appropriate to assess muscle weakness objectively and to detect even very small subclinical changes.

Here, we hypothesized that muscular weakness in patients with CIDP can be objectively quantified with (1) non-invasive quantitative motor (Q-Motor) assessment of grip force and involuntary activity and (2) that pathological findings correlate with disease severity as measured by the MRC and INCAT disability score. In addition, we asked whether patients with atypical manifestation show subclinical signs of generalized muscle weakness compared to healthy controls.

## Methods

### Patients

In this cross-sectional observational study patients who met the diagnostic criteria for typical and atypical CIDP of the EFNS 2010 [9] were included in order to objectively quantify muscular weakness with non-invasive Q-Motor assessment of grip force and involuntary activity. Patients were screened consecutively between March and August 2013 in our outpatient clinic of the Department of Neurology, Charité University Medicine, Berlin, Germany. Data of all CIDP patients was compared to a group of healthy controls (HC) that was similar in age and sex (distribution) to the patient group. HC had to meet the following criteria: Age ≥ 18 years, no other neurological diseases, no other diseases affecting the musculoskeletal system, no cognitive deficits. Overall, 33 patients with CIDP and 28 HC were included. Unlike recent epidemiological data, numbers of recruited typical and atypical CIDP patients were nearly identical for statistical reasons [10]. There was no testing for NF155 and CNTN1 antibodies for patients with atypical CIDP.

### Clinical assessment

Patients were examined by the treating neurologist (JK). Sociodemographics as well as current medication were documented. We used the adjusted inflammatory neuropathy cause and treatment (INCAT) disability score [8] and the Medical Research Council (MRC) sum score [7] to assess the clinical status within 2 months when clinically stable. For the MRC the following muscles had been tested on both sides: shoulder abduction, elbow flexion, wrist extension, index finger extension, hip flexion, knee extension, ankle dorsiflexion, extension of the big toe. For classification, we used CIDP disease activity status (CDAS) [11], summarizing unstable active and improving status as unstable stage, stabile active status and remission status as stable stage..

### Quantitative motor assessment and data analysis

The Quantitative-Motor (Q-Motor) grip-and-lift task (manumotography) providing data for “Quantitative Grip Force Assessment” (QGFA) and “Quantitative Involuntary Movement Assessment” (QIMA) was performed as described earlier [12, 13]. Briefly, all subjects were seated in an upright position. They were then asked to grip and lift an instrument with two force-torque sensors (Nano-40, ATI, Apex, NC), which measured the grip (normal) and load (tangential) forces of the thumb and index finger. The instrument’s weight could be modified to 250 g (light) and 500 g (heavy). An electromagnetic sensor (Fastrack, Polhemus, VT) constantly measured the instrument’s three-dimensional position (x, y, z) and orientation (roll, pitch, yaw). The object was held 10 cm adjacent to a marker for 15 s (static holding phase). Patients performed five consecutive recorded trials with each object weight (light and heavy) and with both hands.

The mean isometric grip force (GF) was calculated as the average of the 5 repetitions during the static holding phase. The amount of involuntary movement during the static holding phase was assessed by recording changes in position (x, y, z) and orientation (roll, pitch, yaw). In order to assess the mean amount of involuntary movements during the static phase, the means of the absolute values of the derivatives of position (the x, y, and z channels) and of the orientation (roll, pitch, and yaw channels) were calculated and summed up to create a position index (PI) and an orientation index (OI) [14]. For patients who were treated with infusions on a regular basis, Q-Motor tests were obtained in the middle of an infusion interval in order to avoid end of dose effects.

All Q-Motor data was transferred to the Q-Motor group at the George-Huntington-Institute, Muenster, Germany, for blinded quality control and automated computerized data analysis. Q-Motor outcomes were then returned to the Charité for further analysis.

### Statistical analysis

Statistical analyses were performed using Prism 6 software (GraphPad, La Jolla, CA, USA). For group differences with regard to sex and treatment history Fisher’s exact test was used. For normally distributed age, MRC and INCAT score, unpaired t-test was used. QGFA and QIMA parameters were tested by the two-tailed Mann-Whitney and Kruskal-Wallis tests followed by Dunn’s multiple comparison tests in two and more groups, respectively. Level of significance was defined as *p* <  0.05 for all comparative tests. We did not perform corrections for multiple comparisons as this was an exploratory study to explore proof-of-concept for quantitative measurements for future research. To detect associations among Q-Motor measures (GF, PI, OI) and disease severity as measured by the MRC-A score and INCAT ODSS, we calculated Spearman coefficients.

## Results

### Patient characteristics

Altogether 33 CIDP patients were included, of these 22 (66.7%) fulfilled EFNS criteria of definite CIDP, 10 (30.3%) patients of probable CIDP and one patient with possible CIDP (3%). Mean age was 64 years (median 65, 44–82) with no significant difference to control group with a mean age of 59 years (median 60, 37–77). There was a huge range regarding disease duration with a minimum of one year and a maximum of 28 years (Median 5, IQR 2–8). CIDP patients and controls did not differ in sex, however atypical and typical patients differed in sex significantly (see Table 1). 16 patients suffered from typical CIDP (48.5%), and 17 patients from atypical variants (51.5%). Of those with atypical variants, 10 patients suffered from DADS, 4 patients of sensory CIDP and 3 of MADSAM (see Table 1). Patients with typical CIDP had a higher INCAT ODSS compared to atypical CIDP (*p* = 0.0004), which was also confirmed for INCAT-A (INCAT Score of the arms) (*p* = 0.003). Mean MRC–sum Score was 71.3 (SD 7.5; Minimum 48, Maximum 80). However, MRC was only obtained from 22 patients. At the time of assessment, 19 (57.6%) patients had been treated with IVIG, 7 (21%) with glucocorticosteroids, none with plasma exchange (PE), 5 (15%) with other immunosuppressants (methotrexate, azathioprine) and two received no treatment. The majority of patients had been treated with glucocorticosteroids sometime before. Only about 24% received plasma exchange in history. There was no difference between typical and atypical CIDP in terms of treatment procedures (Table 1).

**Table 1:** Patient characteristics

	CIDP all	Typical CIDP	Atypical CIDP	HC
*N total*	33	16	17	28
sex, female (%)	12 (36,4)	10 (62,5)	2 (11,76) *P* = 0.004	14 (50)
age (years), mean (SD)	64,18 (9,94)	62,50 (11,65)	65,75 (8,04)	59,54 (8,94)
median (range)	65 (44–82)	61 (44–82)	68 (52–80)	60 (37–77)
time since diagnosis, years, median (IQR)	5 (2–8)	7,5 (2.75–14.25)	3 (2–7)	n.a.
CIDP disease activity status –stabile (%)	26 (85.8)	14 (87.5)	12 (70.6)	n.a.
handedness, right handed (%)	31/32^a^ (96.8)	16 (100)	16 (94)	n.a.
INCAT ODSS, mean (SD)	3.4 (1.6)	4.4 (1.5)	2.4 (1.0) *P* = 0.0002	n.a.
- arms	1.4 (0.75)	2.4 (1.0)	1.4 (0,96) P = 0.003	n.a.
- legs	1.5 (0.9)	2 (1.0)	1.0 (0.001)	n.a.
MRC, mean (SD)	71.5 (7.5)	68.9 (9.9)	73.0 (5.1)	n.a.
treatment actually/ever (%)				
- glucosteroids	7/24 (21/72.8)	5/13 (31/81)	2/11 (12/64)	n.a.
- IVIg	19/33 (58/100)	10/16 (63/100)	9/17 (53/100)	n.a.
- PE	0/8 (0/24.2)	0/4 (0/25)	0/4 (0/23)	n.a.
- other	5/7 (15/21)	3/5 (19/31)	2/2 (12/12)	n.a.

^a^ missing data of one patient regarding handedness in CIDP group

Abbreviations: *INCAT* Inflammatory Neuropathy Cause and Treatment disability score, *MRC* Medical Research Counsil, *SD* Standard deviation, *IQR* Interquartile range, *IVIg* Intravenous immunoglobulin, *PE* Plasma exchange

### Quantitative grip force and involuntary movement assessment in CIDP versus controls

The Q-Motor measures position index (PI), orientation index (OI), and grip force variability (GFV) were significantly increased in all CIDP patients compared to HC for both weights in the left and also in the right hand (Table 2, shown here only data for 500 g). No differences between CIDP patients and HC were found for isometric grip force (for details see Table 2).

**Table 2:** QGFA and QIMA parameters in CIDP versus controls

Q-Motor measure (unit)	CIDP	HC	*p*-value	*n* CIDP/HC
Mean isometric grip force 500g_left hand (*N*)	10.86 (4.24)	9.1 (2.95)	0.1733	32/27
Mean isometric grip force 500g_right hand (*N*)	10.55 (3.95)	10.38 (4.05)	0.8881	31/28
Grip force variability 500g_ left hand (%)	6.97 (2.34)	5.40 (1.79)	0.0091	32/27
Grip force variability 500g_ right hand (%)	6.36 (1.96)	5.4 (2.38)	0.0085	31/28
Orientation index 500g_ left hand (°*/s*)	11.15 (6.09)	5.31 (4.13)	< 0.0001	30/26
Orientationindex 500g_ right hand (°*/s*)	12.19 (7.74)	5.17 (3.36)	< 0.0001	30/27
Position index 500g_ left hand (*cm/s*)	3.50 (1.51)	1.56 (0.83)	< 0.0001	30/26
Position index 500g_ right hand (*cm/s*)	3.49 (1.65)	1.84 (1.47)	< 0.0001	30/27

### Subgroups analysis of atypical versus typical CIDP

There was a statistical difference between typical, atypical CIDP and HC for the position index (PI) as well as for orientation index (OI) (Kruskal-Wallis-test *p* <  0.001). In post hoc analysis, PI as well as OI differed significantly between typical or atypical and HC for both weight classes. However, no statistical difference could be detected between typical and atypical groups itself in post hoc analysis (Table 3). Grip force variability differed between CIDP subgroups and HC for both weight classes with no difference between typical and atypical CIDP in post hoc analysis (Table 3 for 500 g, data not shown for 250 g). Patients with only very mild affection of the arm function (INCAT-A ≤ 1, *n* = 11 [36.7%]) as well as patients with higher arm function disability (INCAT-A ≥ 2, *n* = 21 [63.3%]) showed significantly higher values for the PI as well as for OI for both hands and weights compared to HC. For grip force variability, only patients with INCAT-A ≥ 2 showed significant increased values compared to HC (Table 4 for 500 g, data not shown for 250 g).

**Table 3:** Subgroup analyses of typical and atypical CIDP

Parameter (Unit)	Typical CIDP	Atypical CIDP	HC	*p*-value	Post hoc*	No.**
Mean isometric grip force 500g_left hand (*N*)	9.34 (3.19)	12.20 (4.67)	9.1 (2.95)	0.0777	a) ns b) ns c) ns	15/17/27
Mean isometric grip force 500g_right hand (*N*)	9.40 (3.93)	11.70 (3.73)	10.38 (4.05)	0.1681	a) ns b) ns c) ns	16/16/28
Grip force variability 500g_ left hand (%)	6.94 (2.56)	6.99 (2.20)	5.40 (1.79)	0.0319	a) ns b) ns c) ns	15/17/27
Grip force variability 500g_ right hand (%)	7.16 (2.11)	5.56 (1.46)	5.4 (2.38)	0.0043	a) ** b) ns c) ns	16/16/28
Orientation index 500g_ left hand (°*/s*)	13.24 (7.38)	9.54 (4.47)	5.31 (4.13)	< 0.0001	a) *** b) ** c) Ns	13/17/26
Orientation index 500g_ right hand (°*/s*)	13.45 (6.37)	11.08 (8.82)	5.17 (3.36)	< 0.0001	a) *** b) ** c) Ns	14/16/27
Position index 500g_ left hand (*cm/s*)	3.43 (1.35)	3.59 (1.65)	1.56 (0.83)	< 0.0001	a) *** b) *** a) *Ns*	10/19/26
Position index 500g_ right hand (*cm/s*)	3.20 (1.91)	3.68 (1.56)	1.84 (1.47)	< 0.0001	a) ** b) *** a) *Ns*	10/19/27

* a) Typical CIDP vs. HC, b) Atypical CIDP vs. HC, c) Typical CIDP vs. Atypical CIDP

** typical/atypical/HC

**Table 4:** Subgroup analyses of mild and higher function disability

Parameter (Einheit)	INCAT-A ≤ 1	INCAT-A ≥ 2	HC	*p*-value	Post hoc	No.
Mean isometric grip force 500g_left hand (*N*)	11.44 (4.64)	10.64 (4.18)	9.1 (2.95)	0.3487	a) *ns* b) *ns* c) *ns*	11/20/27
Mean isometric grip force 500g_right hand (*N*)	10.28 (2.47)	10.77 (4.69)	10.38 (4.05)	0.9712	a) *ns* b) *ns* c) *ns*	11/20/28
Grip force variability 500g_left hand (%)	6.55 (2.18)	7.27 (2.47)	5.40 (1.79)	0.0223	a) *ns* b) * c) *ns*	11/20/27
Grip force variability 500g_right hand (%)	5.97 (2.35)	6.63 (1.77)	5.4 (2.38)	0.0123	a) *ns* b) ** c) *ns*	11/20/28
Orientation index 500g_left hand (°*/s*)	10.24 (4.02)	11.78 (7.08)	5.31 (4.13)	< 0.0001	a) ** b) *** c) *ns*	10/19/26
Orientation index 500g_right hand (°*/s*)	10.46 (9.32)	13.17 (7.09)	5.17 (3.36)	< 0.0001	a) * b) *** c) *ns*	10/19/27
Position index 500g_left hand (*cm/s*)	3.43 (1.35)	3.59 (1.65)	1.56 (0.83)	< 0.0001	c) *** d) *** e) *ns*	10/19/26
Position index 500g_right hand (*cm/s*)	3.20 (1.91)	3.68 (1.56)	1.84 (1.47)	< 0.0001	c) ** d) *** e) *ns*	10/19/27

* a) INCAT ≤1 vs. HC, b) INCAT ≥2 vs. HC, c) INCAT ≤1 vs. INCAT ≥2

** INCAT-A ≤ 1/ INCAT ≥2/ HC

### Correlation with INCAT ODSS and MRC

**Fig. 1:**
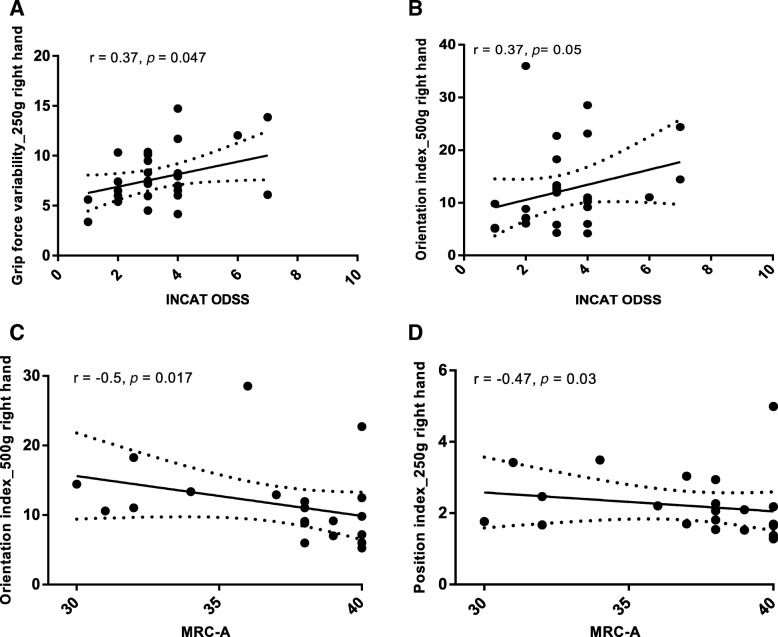
Q-Motor involuntary movement measures correlations with CIDP disability scores. Correlations of grip force variability (**a**) position and orientation index with INCAT disability score for the right hand heavy (**a**) and light weight (**b**). Correlations of orientation index of the right hand for 500 g (**c**) as well as position index of the right hand (250 g) with MRC-A (D). p = *p*-value, r = Pearson correlation coefficient, solid line = fit line, dotted line = 95% CI

For all parameters that were significantly increased in CIDP patients, we assessed the correlation with the INCAT and MRC score. There was a weak correlation of GFV right hand 250 g of all CIDP patients with INCAT score (Fig. 1a, Spearman correlation index r = 0.37, *p* = 0.047) but not with MRC (data not shown). OI of both hands correlated by trend with INCAT (Fig. 1b shows for the right hand, Spearman correlation index r = 0.37, *p* = 0.05). There was also a moderate correlation of OI with MRC-A of both hands for 500 g (Fig. 1c shows right hand, for the left hand: *r* = − 0.43, *p* = 0.04) and for the right hand with 250 g (*r* = − 0.47, *p* = 0.03). There was no correlation of PI with INCAT or INCAT-A (data not shown). However, we found a moderate correlation of PI with MRC-A for the both hands (Fig. 1d shows 250 g right hand, for the left hand *r* = − 0.46; *p* = 0.04; 500 g *r* = − 0.47, *p* = 0.03).

## Discussion

In this cross-sectional pilot study we investigated 33 CIDP patients compared to 28 HC using the Q-Motor grip-and-lift task quantitatively assessing grip force and involuntary movements. Aim of the study was to objectively assess muscle weakness and to detect even very small subclinical changes in CIDP patients as well as to search for differences between typical and atypical CIDP variants. Involuntary movements parameters such as GFV, OI and PI were significantly elevated in CIDP compared to HC. Subgroup analysis revealed no difference between typical and atypical CIDP groups itself. Interestingly, patients with only very mild affection of the arms (INCAT-A ≤ 1) showed the same alterations of OI and PI compared to more disabled patients (INCAT-A ≥ 2).

Beside sensory disturbance, the assessment of muscular weakness is essential in order to establish diagnosis as well as evaluate treatment outcome.

So far, MRC and Martin’s Vigorimeter are established endpoints applied in clinical routine. Grip strength can be measured by using the Vigorimeter, a simple objective tool that is easy to apply [15, 16]. However, it is only a one-dimensional tool and thus may not be sensitive enough to measure small subclinical changes in manual coordination, for instance at disease onset or in atypical variants.

The medical research council scale (MRC) also works on the impairment level but has a great interrater variability and thus inherited imprecision. As a third outcome measure, the INCAT disability scale assesses disabling problems in daily arm and leg mobility and has evolved as the most established primary outcome in clinical trials since it was used in the large ICE trial of CIDP patients [17, 18]. However, it mainly detects actual disability and by definition is not sensitive to detect smaller alterations.

The quantitative Q-Motor assessments were shown to detect subclinical changes in patients with premanifest Huntington’s disease and correlated well with disease severity [13, 14, 19] and progression even 2 decades before disease onset [20, 21]. Q-Motor measures detected drug effects not seen in rater-dependent categorical clinical scales in several multicenter clinical trials [22, 23]. Also, Q-Motor testing has been proven as a meaningful tool in order to measure muscular weakness in myasthenia gravis [24].

In the present study we show significantly elevated QIMA parameters in CIDP compared to HC. Subgroup analysis showed elevated OI and PI in atypical and typical CIDP compared with HC with no difference between both patient groups suggesting a good applicability also for atypical variants. Although the clinical picture differs markedly between CIDP subgroups, no differentiation has been performed in published studies [16, 18] when using the INCAT score or Martin vigorimeter. QIMA parameters may be a meaningful tool in order to quantify muscular weakness also in atypical variants, which needs to be investigated in a larger multicenter study. In contrast to OI and PI, mean grip force did not differ significantly in typical or atypical CIDP from HC. We therefore concluded that involuntary movement parameters might be more sensitive and thus more suitable in order to detect even small changes in muscular weakness. Accordingly, stratification in patients with only mild affection of arm function (INCAT-A ≤ 1) showed significantly higher values for the PI as well as for OI for both hands and weights compared to HC.

In particular, patients with atypical manifestation are misdiagnosed very frequently in almost 50% of all cases [4] based on missing validated diagnostic criteria which have only very recently described in more detail [10]. This leads to unnecessary and costly treatment; a recent study reported that only 7 of 65 patients, i.e. 11%, receiving long-termly intravenous immunoglobulins actually fulfilled the EFNS diagnostic criteria of CIDP [25]. Application of objective and sensitive assessments of symptom severity and treatment efficacy may help to reduce misdiagnosis and overtreatment.

We acknowledge several limitations of this study. Although the sample size of 33 CIDP patients is comparatively large considering that CIDP is an orphan disease it is still too small to establish QIMA parameters as diagnostic and/or efficacy parameters in CIDP subgroups. This would need further confirmatory studies. MRC hasbeen obtained only from 22 patients. However, despite the limited number of MRC measurements, correlation with QIMA parameters reached statistically significance. In addition, grip strength parameters by Martin vigorimeter or Jamar [16, 26] have not been obtained and should be included in a further larger cohort trial.

Recently, Knak et al. showed that grip strength is less responsive to detect improvements after treatment than isokinetic dynamometry at ankles in a retrospective study. The authors concluded that grip strength as measured by JAMAR dynamometer does not seem to be an appropriate surrogate parameter of overall muscle strength and should be combined with isokinetic measurements of the lower limbs [27]. In the present study, we did not search for treatment efficacy parameters. However, in order to obtain appropriate overall muscle strength additional strength parameters of the lower limbs such as isokinetic measurements or foot tapping by Q-Motor may have to amended. Furthermore, we did not perform corrections for multiple comparisons, as this was an exploratory proof-of-concept study. However, the changes in OI and PI were robust and consistent, supporting a likely biological relevance of our findings. In addition, almost all patients had a stable disease activity status, i.e. they were stable under treatment or in remission. Measuring treatment naïve patients in larger cohort trials would probably enhance the observed effects.

## Conclusions

Q-Motor tests may be useful to objectively assess muscular weakness in CIDP. The observations of our study encourage further exploration in larger cohort trials. In particular, QIMA parameters seem to be suitable to detect subclinical generalized muscle weakness in patients with milder disability and/or atypical phenotype. Therefore, they might be a sensitive and reliable endpoint in future CIDP trials and support data-driven, unbiased decision making in clinical development of novel therapies.

## Data Availability

The datasets used and analysed during the current study are available from the corresponding author on reasonable request.

## Abbreviations

AAN: American Academy of Neurology
CDAS: Clinical disease activity score
CIDP: Chronic inflammatory demyelinating polyneuropathy
DADS: Distal acquired demyelinating polyneuropathy
EFNS: European Federation of Neurological Societies
GF: Grip force
GFV: Grip force variability
HC: Healthy control
INCAT: Inflammatory neuropathy cause and treatment
IQR: Interquartile range
IVIg: Intravenous immunoglobulin
MADSAM: Multifocal acquired demyelinating sensory and motor polyneuropathy
MRC: Medical Research Counsil scale
OI: Orientation index
PI: Position index
QGFA: Grip Force Assessment
QIMA: Involuntary Movement Assessment
Q-Motor: Quantitative motor test
SD: Standard deviation
